# Building Ultra-Stable and Low-Polarization Composite Zn Anode Interface via Hydrated Polyzwitterionic Electrolyte Construction

**DOI:** 10.1007/s40820-022-00835-3

**Published:** 2022-04-06

**Authors:** Qiong He, Guozhao Fang, Zhi Chang, Yifang Zhang, Shuang Zhou, Miao Zhou, Simin Chai, Yue Zhong, Guozhong Cao, Shuquan Liang, Anqiang Pan

**Affiliations:** 1 School of Materials Science and Engineering, Key Laboratory of Electronic Packaging and Advanced Functional Materials of Hunan Province, Central South University, Changsha, 410083 People’s Republic of China; 2 Joint School of National University of Singapore and Tianjin University International Campus of Tianjin University Binhai New City, Fuzhou, 350207 People’s Republic of China; 3 Department of Materials Science and Engineering, University of Washington, Seattle, WA 98195 USA

**Keywords:** Quasi-solid electrolyte interface, Polyzwitterionic hydrogel electrolytes, High performance, Manganese dioxides, Zinc metal anodes

## Abstract

Aqueous zinc metal batteries are noted for their cost-effectiveness, safety and environmental friendliness. However, the water-induced notorious issues such as continuous electrolyte decomposition and uneven Zn electrochemical deposition remarkably restrict the development of the long-life zinc metal batteries. In this study, zwitterionic sulfobetaine is introduced to copolymerize with acrylamide in zinc perchlorate (Zn(ClO_4_)_2_) solution. The designed gel framework with hydrophilic and charged groups can firmly anchor water molecules and construct ion migration channels to accelerate ion transport. The in situ generated hybrid interface, which is composed of the organic functionalized outer layer and inorganic Cl^−^ containing inner layer, can synergically lower the mass transfer overpotential, reduce water-related side reactions and lead to uniform Zn deposition. Such a novel electrolyte configuration enables Zn//Zn cells with an ultra-long cycling life of over 3000 h and a low polarization potential (~ 0.03 V) and Zn//Cu cells with high Coulombic efficiency of 99.18% for 1000 cycles. Full cells matched with MnO_2_ cathodes delivered laudable cycling stability and impressive shelving ability. Besides, the flexible quasi-solid-state batteries which are equipped with the anti-vandalism ability (such as cutting, hammering and soaking) can successfully power the LED simultaneously. Such a safe, processable and durable hydrogel promises significant application potential for long-life flexible electronic devices.

**Supplementary Information:**

The online version contains supplementary material available at 10.1007/s40820-022-00835-3.

## Highlights

- A novel hydrogel with high water retention and Zn^2+^ transference number of 0.604 was constructed by copolymerizing sulfobetaine and acrylamide in Zn(ClO_4_)_2_ solution.
- The designed electrolyte configuration enables in situ generation of the organic–inorganic hybrid interface, which contributes to the electrodeposition uniformity and corrosion resistance of the anode.
- Zn–Zn and Zn–MnO_2_ cells based on hydrogel electrolyte exhibit outstanding cycling stability (over 3000 h under 0.5 mA cm^−2^/0.5 mAh cm^−2^ after two-time shelving).

**Supplementary Information:**

The online version contains supplementary material available at 10.1007/s40820-022-00835-3.

## Graphical abstract

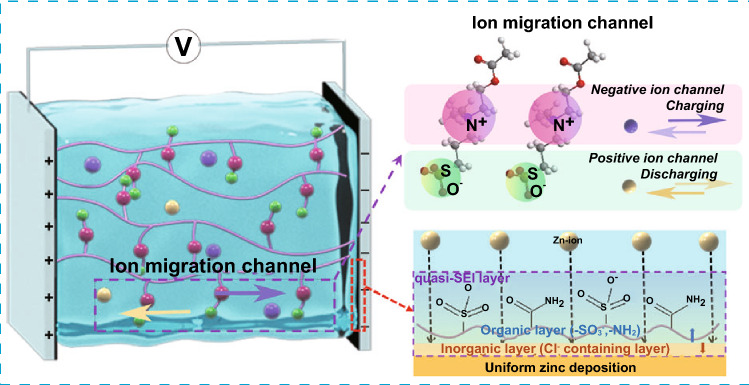

## Introduction

Numerous research on batteries with environmental friendliness, intrinsic safety and satisfactory energy densities has been conducted in the field of energy storage systems [1–8]. Of the various developed batteries, aqueous zinc metal batteries (ZMBs) are the most competitive and promising candidate to be applied as the commercially viable energy storage device with a low electrochemical potential of − 0.763 V (vs. SHE), relatively high volume capacity (5855 mAh cm^−3^), high safety and benign water compatibility [9–13]. However, some long-lasting tricky issues such as dendrite traits and side reactions severely affect the lifespan of the aqueous ZMBs [14–18]. Various effective strategies have been exploited to alleviate these undesirable issues, exemplified with artificial interface modification [19], structural optimization [20–24], electrolyte regulation [25–33] and using gel electrolytes [34–36].

The great triumph achieved in ZMBs based on the hydrogel electrolyte was benefited from the satisfactory flexibility and wearability, induced by the relatively low-content interstitial water and the robust cross-coupling network in the hydrogel. Besides, the gel electrolyte can also increase the energy density of the battery due to its excellent water retention and broadened electrochemical window, and ultimately greatly prolong the service life of flexible Zn-based devices. Previously, poly(vinyl alcohol) (PVA) [37–39], polyacrylamide (PAM) [40–43] and polysaccharide (gelatin [44, 45], alginate [46, 47], etc.) have emerged as crucial categories of the gel electrolytes community for aqueous ZMBs due to their rich hydrophilic groups. Unfortunately, although the hydrogel electrolyte, to some extent, can inhibit some side reactions with zinc anode due to less water content, the unfavorable interface electrochemistry between the anode and hydrogel electrolyte is still regarded as the main reason for the shortened battery life. One typical interface optimization strategy is constructing an artificial solid electrolyte interface (SEI), which can effectively alleviate electrode deterioration and electrolyte decomposition. However, the artificial SEI currently suffers from unavoidable cracks during the cycles [14, 48], poor interface contacts with Zn and dissatisfactory ion conductivity, all of which would lead to limited inhibition of dendrites and unfavorable Coulombic efficiencies (CEs). Very recently, highly concentrated electrolytes (e.g., “water-in-salt”) [28, 49] and multiple functional additives [50, 51] were introduced into ZMBs to improve the CEs and extend their life spans. The high costs involved and unstable/ineffective SEI protective layer, however, severely hinder their future developments. Combining the flexibility advantages of hydrogel with the interphase modification is expected to solve those tricky/undesirable issues simultaneously and achieve increased battery life.

Herein, a betaine-type zwitterionic monomer 3-((2-(methacryloyloxy)ethyl)dimethylammonio) propane-1-sulfonate (DMAPS) was introduced to copolymerize with acrylamide (AM) to further improve the electrochemical performance of the hydrogel electrolyte [52–54]. Owing to the zwitterionic nature and the high hydration ability of the charged groups within the hydrogel electrolyte, ions can be easily separated from the counterions, leading to the accelerated ion migration and enhanced rate performance. Meanwhile, the hydrogel electrolyte delivers high water retention; there is a certain degree of self-healing and mechanical properties due to the interaction between the hydrophilic groups and water molecules, the hydrogen interaction and the “zwitterionic fusion,” respectively [55, 56]. Furthermore, the intrinsic hydrophilic and charged groups in the polyzwitterion chain can guide the uniform transportation of zinc ions and consequently lead to dendrite-free deposition. What is more, replacing the conventionally used ZnSO_4_ with Zn(ClO_4_)_2_ salt contributes to the in situ formed Cl^−^ containing insulating layer upon Zn metal, which consequently lead to ZMB with low polarization and ultra-long cycle life [26, 57]. The designed gel electrolyte with Zn(ClO_4_)_2_ salt (denoted as ADC-gel) inherits the advantages of the zwitterionic copolymer and the selected salt solution to synergically construct the compatible composite quasi-SEI layer, which could remarkably pave the way for the anti-corrosion and the high cycle reversibility of the ZMBs. Zn//Zn cells based on ADC-gel electrolytes offers extremely long cycle lives: more than 3000 h at the current density of 0.5 mA cm^−2^and 800 h at an even much higher current density of 5 mA cm^−2^. Superior rate capability with low polarization voltage (below 0.05 V) under a wide range of current densities (from 0.5 to 5 mA cm^−2^) was also achieved. When matched with MnO_2_ cathode, ZMB full cell could deliver a high capacity of 350 mAh g^−1^ and maintain more than 100 cycles without obvious capacity decay. To assess the practicality of the fabricated hydrogel, pouch cells can work normally in driving the practical devices under harsh working conditions, including cutting, soaking, hitting, etc.

## Experimental Section

### Materials Synthesis

#### Synthesis of ADC-gel Electrolyte and Liquid Electrolyte

The designed polyzwitterion hydrogel was fabricated by a thermal-initiated method as follows: first, dispersed a certain amount of monomers (0.75 g acrylamide (AM, Aladdin, AR) and 0.75 g 3-dimethyl(methacryloyloxyethyl) ammonium propane sulfonate (DMAPS, Aladdin)) in the 10 mL 2 M zinc perchlorate Zn(ClO_4_)_2_ solution, where the zinc perchlorate (Zn(ClO_4_)_2_·6H_2_O) was also got from Aladdin. Initiator potassium persulfate (K_2_S_2_O_8_, Sinopharm Chemical Reagent) relative to 0.5% total monomers mass and cross-linking agent *N*,*N*'-methylenebisacrylamide (MBAA, Macklin) relative to 0.4% total monomers mass were subsequently added into the above-mixed solution. After the vigorous stirring, a degassing procedure was indispensable and performed through purging nitrogen gas into the transparent solution for minutes. The thin hydrogel film of around 1 mm was obtained by transferring the as-prepared solution into a handmade glass mold by a pipettor and performing the sol–gel transition at 80 °C for 12 h. When matched with MnO_2_ cathode, the precursor solution needed to add additional MnSO_4_·H_2_O (Sinopharm Chemical Reagent) before the free radical polymerization process. For the liquid electrolyte, added 3.7238 g Zn(ClO_4_)_2_ to 20 mL deionized water and then transferred this mixed solution to a 50 mL volumetric flask. The above solution was diluted to volume with deionized water to acquire 2 M Zn(ClO_4_)_2_ aqueous solution.

#### Synthesis of Cathodes

A traditional hydrothermal synthesis method was employed to obtain MnO_2_ cathode. Generally, a certain amount of MnSO_4_^.^H_2_O was added to 15 mL deionized water to get 0.15 M MnSO_4_ solution and then added 0.237 g KMnO_4_ in 15 mL deionized water with stirring. MnSO_4_ solution was added into the KMnO_4_ solution drop by drop to ensure an adequate oxidizing environment. Subsequently, the final prepared solution  was transferred into a Teflon-lined autoclave and heated in the oven for 12 h at 160 °C. After centrifugation several times with washing and drying under 60 °C, the MnO_2_ powder was collected. The MnO_2_ cathode was fabricated by mixing the resultant MnO_2_, conductive agent Super P and binder polyvinylidene fluoride (PVDF) in a weight ratio of 7:2:1 in NMP solvent and then blade coating the mixture on the stainless steel mesh with a diameter of 12 mm. After subsequently drying in a vacuum oven at 80 °C for 12 h, the MnO_2_ cathode was finally got. The V_2_O_5_ (Aladdin, 99.0%) was purchased to fabricated the cathode, and the preparation method was the same as that of the MnO_2_ cathode.

### Characterization

Fourier transform infrared spectrometer (FTIR, AVTATAR, 370 with the frequency range of 500–4000 cm^−1^) was employed to analyze the functional groups or chemical bonds of monomers and polymers. The surface morphology of the Zn anode after cycles was characterized by the scanning electron microscope (SEM) measurement using Nova Nano SEM 230 and MIRA3 LMH[YZ1]. The energy-dispersive spectrometer (EDS, NiuJin X MAX20) was selected to investigate the element component and distribution on the anode surface. The X-ray photoelectron spectroscopy (XPS) analysis was carried out by ESCALAB 250 Xi X-ray photoelectron spectrometer (Thermo Fisher). The phase composition of the by-products coated on the anode surface was characterized by X-ray diffraction measurement (XRD, Rigaku, D/Max-2500 with Cu K*α1* radiation, *λ* = 1.5418 Å). The in situ optical observation was carried out by the transflective polarizing microscope LW750LJT to evaluate the interface compatibility in the different electrolytes.

### Electrochemical Measurements

CR2032 coin cells were assembled to measure the electrochemical performance. Cyclic voltammetry (CV) curves, ionic conductivities and ion transference number tests were measured via electrochemical workstations (CHI660D, China and Admiral Instruments). The galvanostatic cycling tests of zinc symmetric cells and Zn–MnO_2_ full cells were conducted in a Neware workstation at constant room temperature.

Stain steel/ADC-gel/Stain steel and Zn/ADC-gel/Zn cells were assembled to investigate ionic conductivities and Zn^2+^ transference number ($t_{{{\text{Zn}}^{2 + } }}$), respectively, via electrochemical impedance spectroscope with a frequency range of 100 kHz to 10 mHz. The ionic conductivities (*σ*) is calculated by Eq. (1):

1 $$\sigma = \frac{d}{RS}$$

where *d* (cm) and *S* (cm^2^) represent the thickness and test area of the as-obtained hydrogel electrolyte, respectively. *R* points to the bulk resistance from the AC impedance spectroscopy.

The Zn^2+^ transference number ($t_{{{\text{Zn}}^{2 + } }}$) of hydrogel electrolyte was characterized by combining the chronoamperometry (CA) technology and EIS method, and derived according to Eq. (2):

2 $$t_{{Zn^{2 + } }} = \frac{{I_{s} \left( {\Delta V - R_{0} I_{0} } \right)}}{{I_{0} \left( {\Delta V - R_{s} I_{s} } \right)}}$$

where $I_{0}$ and $I_{s}$ correspond to the initial and stable state of the current, respectively; $\Delta V$ is the applied amplitude of 5 mV; and $R_{0}$ and $R_{s}$ refer to the electrolyte/electrode interface resistance before and after DC polarization, respectively.

The electrochemical stable window of the hydrogel electrolyte was detected by CV measurement with a scan rate of 0.5 mV s^−1^ between − 0.2 and 2 V (vs. Zn^2+^/Zn) by sandwiching hydrogel electrolyte between Zn anode and stainless steel.

## Results and Discussion

### Formulation and Characterization of ADC-gel Electrolyte

**Fig. 1:**
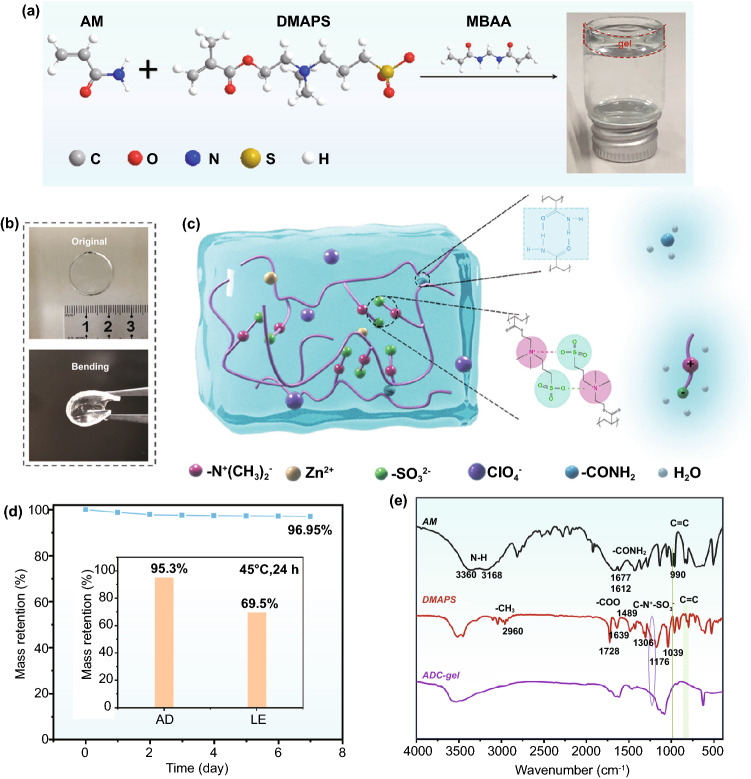
**a** Schematic diagram of the synthesis path of the ADC-gel under thermal initiation. **b** Optical images of the ADC-gel electrolyte with bending characteristic. **c** Schematic of the interaction in the ADC-gel matrix. **d** Mass retention capacity of the ADC-gel when exposed to different temperatures under ambient environment. **e** FTIR spectra of the monomers (AM and DMAPS) and the hydrogel electrolyte

As shown in Figs. 1a and S1, two monomers were copolymerized in the salt solution to form ADC-gel after heating. Two monomers AM and DMAPS were added to Zn(ClO_4_)_2_ solution. With the presence of the initiator KPS and the cross-linker MBAA, the ADC-gel electrolyte was prepared by free radical polymerization. The processed hydrogel electrolyte had a diameter of 19 mm and a thickness of about 1.2 mm and could be bent at will (Figs. 1b and S2).

The prepared hydrogel was scrutinized into inner character, as illustrated in Fig. 1c. Due to the chemically cross-linked rigid copolymer backbones and the relatively weak bond interactions (such as the hydrogen bonds and electrostatic interactions) for energy dissipation, the synthesized hydrogel had certain mechanical properties, such as the bending characteristic. The self-healing ability was validated by the reconnection of the cutting surface and the stretching test of the gel (Fig. S3), which was originated from the interactions between these functional groups. Owing to the interactions between water and the hydrophilic groups (polar –NH_2_ groups and the charged groups), the highly hydrated polymer branches surrounded by the water molecules endow the ADC-gel with a great water retention ability. As shown in Fig. 1d, the hydrogel could maintain 96.95% of its pristine mass at room temperature after a week. After being placed in a 45 °C oven for 1 day, ADC-gel could still preserve more than 95% mass retention, while near 30% of the mass in the liquid electrolyte was evaporated. Fourier transform infrared spectra (FTIR) of AM, DMAPS and the copolymer ADC-gel were recorded to investigate the polymerization mechanism of the hydrogel (Fig. 1e). The initial AM shows strong absorption bands at 3660 and 3168 cm^−1^ due to the N–H stretching vibration. The characteristic bands of the –CONH_2_ group situated at 1677 and 1612 cm^−1^ correspond to C=O stretching and N–H in-plane bending, respectively. As for the structure of the other monomer DMAPS, an obvious absorption at around 3500 cm^−1^ is ascribed to O–H (originated from the absorbed water). Characteristic signals of DMAPS can be assigned as follows: 1728 and 1639 cm^−1^ belong to –COO^−^ group; 1489 cm^−1^ is attributed to C–N^+^ vibration; and two peaks at 1039 and 1176 cm^−1^ are ascribed to –SO_3_^−^ group. Meanwhile, after polymerization, the electrostatic effect between N^+^ and –SO_3_^−^ greatly weaken the intensity of the C–N^+^ bond (1306 cm^−1^), which verifies the presence of electrostatic interactions. In the fingerprint region, the absorption band C=C group of monomers AM and DMAPS at 990 and 810–840 cm^−1^ (marked in green) disappears in the copolymer, demonstrating the successful polymerization. Even if there exhibit a superposition of two monomer spectra and a small frequency shift, characteristic peaks discussed above can still be verified in ADC-gel, which proves the completed preparation of ADC-gel.

### Electrochemical and Ion Transport Properties of the ADC-gel Electrolyte

**Fig. 2:**
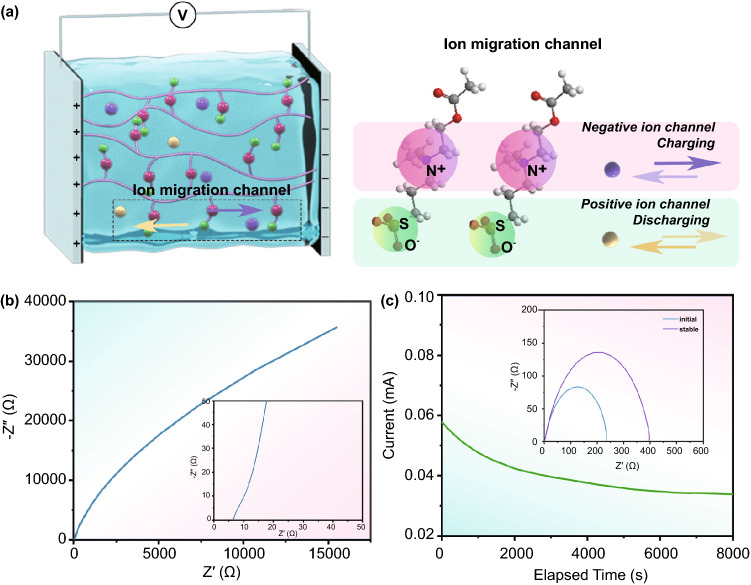
**a** Schematic illustration of the construction of ion migration channel under the applied electric field. **b** EIS spectra of the ADC-gel with two blocked electrodes (stain steels). **c** EIS spectra and the current variation with the polarization of Zn/ADC-gel/Zn symmetric cell

Concerning the molecular structure, ADC-gel is well designed by ushering in zwitterionic components, including negatively charged sulfonate group and positively charged quaternary ammonium group, on the molecular chain. As shown in Fig. 2a, the synthesized polymer backbone is randomly distributed in the gel electrolyte, but when under the action of an external electric field, the ion migration channels parallel to the external electric field can be formed along the arranged zwitterionic side groups [52, 53]. There is a hydration layer along the zwitterionic chains due to the strong electrostatic interactions between charged groups and water molecules [58, 59]. Subsequently, the dissolved electrolyte ions (cationic Zn^2+^ and anionic ClO_4_^−^) in the quasi-solid-state electrolyte can be readily separated from the charged groups under the external electric field without overcoming energy barrier produced by the strong electrostatic attractions [53, 60]. Hence, such ion migration channel accelerates the ion transport efficiency in the ADC-gel polymer electrolytes. The ionic conductivity of the ADC-gel was measured by AC impedance spectroscopy in Fig. 2b. The successful construction of the fast ion transport channels is evidenced by the ion conductivity of 6.48 mS cm^−1^ which is higher than that of many reported hydrogel electrolytes [44, 61]. Particularly, a high ion transference number ($t_{{{\text{Zn}}^{2 + } }}$) is essential for not only the low polarization due to the construction of the smaller concentration gradient, but also the homogeneous Zn deposition and the high capacity of ZMBs. The calculated $t_{{{\text{Zn}}^{2 + } }}$ of the ADC-gel electrolyte in Fig. 2c is 0.604, which is much higher than that of the liquid electrolyte (0.173, Fig. S4). The promoted Zn^2+^ ion migration of the gel electrolyte may be explained by the tethered ion chemistry: the charged groups facilitate the dissociation of Zn^2+^ and the ClO_4_^−^ and the shuttle of Zn ion [62]. Furthermore, the electrochemical stability windows of the electrolytes were also investigated by the linear sweep voltammetry (LSV) method, and the results are shown in Fig. S5. The ADC-gel electrolyte expands the oxygen evolution potential from 2.58 to 2.72 V. The results of the negative scans demonstrate that the hydrogen evolution of ADC-gel is suppressed, and the onset potential dropped from − 0.11 to − 0.19 V. The ADC-gel electrolyte extends the hydrogen and oxygen evolution potentials beyond the thermodynamic stability limits of liquid electrolyte, demonstrating the lowered water activity because of a hydration effect.

### Electrochemical Testing of the Zn Ion Deposition

**Fig. 3:**
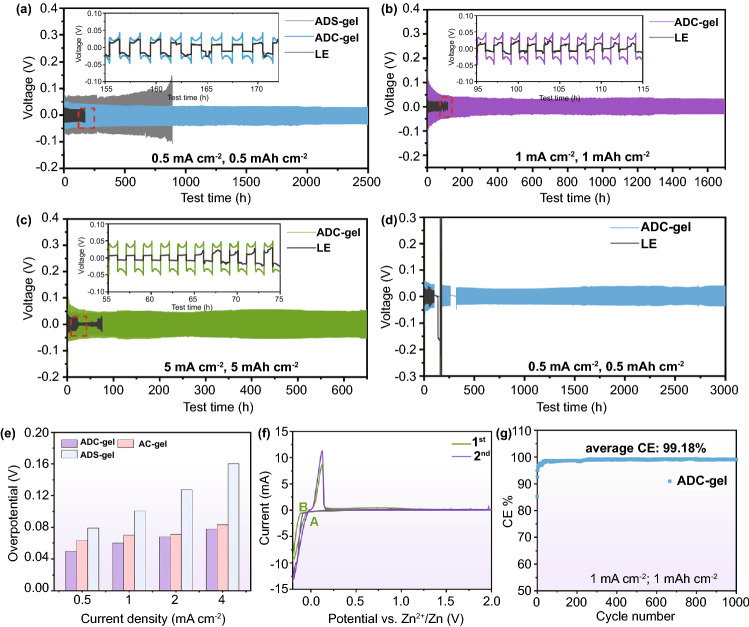
Cyclic stability of symmetrical cells assembled with ADC-gel and liquid electrolytes under current densities and capacities of **a** 0.5 mA cm^−2^, 0.5 mAh cm^−2^, **b** 1 mA cm^−2^, 1 mAh cm^−2^ and **c** 5 mA cm^−2^, 5 mAh cm^−2^. **d** Shelving performance of Zn//Zn symmetrical cells at 0.5 mA cm^−2^, 0.5 mAh cm^−2^. **e** Overpotential profile with different current densities (0.5, 1, 2 and 4 mA cm^−2^). **f** CV curves of Zn anode dissolution and deposition in Zn/ADC-gel/stain steel cell at the scan rate of 0.5 mV s^−1^. **g** CE curve of ADC-gel electrolyte in Zn//Cu asymmetric cell at 1 mA cm^−2^, 1 mAh cm^−2^

**Fig. 4:**
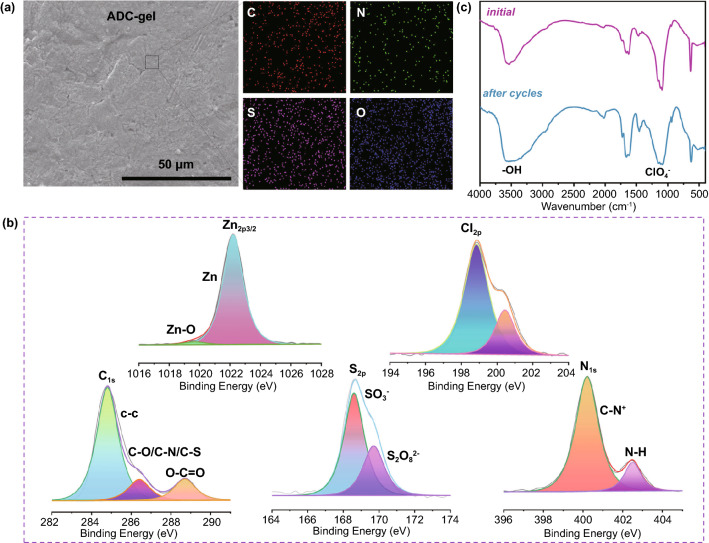
**a** SEM image of the Zn anode in ADC-gel after 60 cycles and the corresponding EDS images of the C, N, S, O elements. **b** High-resolution XPS spectra of the surface of the cycled Zn anode. **c** FTIR spectra of the initial state of the ADC-gel and the surface layer of the zinc anode after cycled in the gel electrolyte

The cycle stability of the Zn anode and the interfacial compatibility between the anode and the gel electrolyte were investigated by galvanostatic cycling of the Zn/ADC-gel/Zn symmetrical cell (Fig. 3a–c). The Zn//Zn symmetrical cell with ADC-gel electrolyte manifests ultra-long cycling life, which can stabilize over 2500 h with a low polarization voltage of ~ 0.03 V and negligible by-products (Fig. S6) at 0.5 mA cm^−2^ and 0.5 mAh cm^−2^. Nevertheless, the Zn//Zn symmetrical cell assembled with Zn(ClO_4_)_2_ aqueous electrolyte suffers a sudden short circuit after cycling for only 160 h (the inset of Fig. 3a). Characterized by good fluidity and low viscosity, the liquid electrolyte has relatively high ion conductivity and fast kinetics on the electrode (Figs. S7 and S4). However, due to the high viscosity of polymer electrolytes, the ionic conductivity of these electrolytes is often hindered, sacrificing ion transport kinetics to some extent [63]. Thus, the polarization of Zn//Zn cells with ADC-gel is higher than that with liquid electrolyte. The ZnSO_4_ salt was selected to replace the Zn(ClO_4_)_2_ salt in the hydrogel matrix to form ADS-gel. It is worth noting that the ADS-gel electrolyte exhibits a much higher polarization voltage than that of the ADC-gel electrolyte-based cell and experiences increasing voltage polarization as the cycles go on. The relatively short cycling life of ADS-gel electrolyte-based cell is attributed to the chaotic and relative incompatible dendrite surface (Fig. S8). But the gel electrolyte still extends the cycling life of the zinc sulfate aqueous electrolyte (Figs. S9 and S10). When increasing the current density to 1 and 5 mA cm^−2^, high reversible plating/stripping efficiencies can be achieved even after 1700 and 700 h cycles, respectively, in ADC-gel electrolyte-based cells, indicating the uniform ion distribution, robust and compatible interface. Average polarization slightly increases (corresponding to 0.032 and 0.05 V, respectively) with increasing current densities, whereas the cell assembled with 2 M Zn(ClO_4_)_2_ aqueous electrolyte shows premature failure after only 80 h at 1 mA cm^−2^ and 20 h at 5 mA cm^−2^. The Zn//Zn symmetrical batteries were employed to demonstrate the shelving performance as well (Fig. 3d). The battery based on the aqueous electrolyte has failed after one period of 60 h shelving. Apparently, although a subtle decrease is observed, no visible voltage increases can be found after the first 60 h shelving when cycled with ADC-gel electrolyte. Besides, the designed electrolyte allows the battery with exceptional cycle stability nearly 3000 h after the second 60 h shelving. The electrochemical performance is superior to many other reported hydrogel electrolytes (Table S1). SEM images (Figs. 4a and S12a) show a smooth and dendrite-free morphology of Zn metal anode after 60 plating/stripping cycles in ADC-gel electrolyte at 0.5 mA cm^−2^, which further verify the uniform Zn deposition favorably tailored by the employment of ADC-gel electrolyte. Conversely, in the liquid electrolyte, the zinc anode with chaotic surface fluctuation is specifically presented in quite many micro-sized plates with sharp edges and bulk aggregations, indicating the formation of detrimental by-products and the pulverizing of Zn deposits (Figs. S11 and S12b).

Furthermore, to investigate the electrochemical behavior of Zn anode in ADC-gel electrolyte, the polarization voltage of Zn stripping and plating at a wide range of current densities from 0.5 to 4 mA cm^−2^ is shown in Fig. 3e. The gel electrolyte (with or without DMAPS monomer) and salt additives (Zn(ClO_4_)_2_ or ZnSO_4_) act as variables to evaluate their rate capability, respectively. What can be intuitively found is that neither the ADS-gel electrolyte nor the AC-gel electrolyte (PAM hydrogel with Zn(ClO_4_)_2_ salt) has a higher polarization voltage than the ADC-gel electrolyte. It is worth noting that the ADC-gel not only enables a much lower polarization voltage than the ADS-gel, but also delivers a very slight increase in overpotential during cycling. One possible reason is that the highly water-soluble nature of Zn(ClO_4_)_2_ salt decreases the viscosity and increases the conductivity of the gel electrolyte (Figs. 2b and S13) [64]. Another reason is that the electrically insulating but Cl^−^ containing ion-conducting layer generated in situ during the cell cycling is conducive to the transportation of zinc ions and result in reduced interface resistance (Fig. S14) [26]. The comparison of ADC-gel and AC-gel proves that the introduction of zwitterionic DMAPS in AM can accelerate rapid ion migration because of the low concentration gradient constructed by the polyzwitterion channel. Cyclic voltammetry (CV) was conducted to evaluate the reversible process of the Zn deposition and dissolution in ADC-gel electrolyte and liquid electrolyte. As exhibited in Figs. 3f and S15, the potential difference between the points of intersection A and B corresponds to the nucleation overpotential (NOP), and ADC-gel electrolyte elevates the NOP from 68 (AB_LE_) to 118 mV (AB_ADC-gel_). CE is another considerable index to evaluate the reversibility and endurability of metallic electrodes in the Zn//Cu coin cells. For the 2 M Zn(ClO_4_)_2_ aqueous electrolyte, Zn/Zn^2+^ chemistry reaches the maximum CE of 98% under the current density of 1 mA cm^−2^ and the capacity of 1 mAh cm^−2^, but it starts to decline and fluctuate and finally short-circuited after 65 cycles (Fig. S16). For comparison, the cell with the gel electrolyte quickly reaches a CE of around 99.18% and maintains stable performance over 1000 cycles. Figure S17 illustrates that the ADC-gel has a greater nucleation overpotential than the liquid electrolyte, which is consistent with the NOP in CV curves (Figs. 3f and S15). Nevertheless, the deposits in the 2 M Zn(ClO_4_)_2_ solution tend to evolve into dendrites due to the “tip effect” [65]. For the galvanostatic cycling test with Cu foil as a working electrode, a small hysteresis of ~ 28 mV but a quick capacity decay is observed during Zn stripping/plating process under the same condition in the aqueous electrolyte (Fig. S18). Although the voltage hysteresis of Zn//Cu cell with ADC-gel electrolyte is around 43 mV, the extremely stable and reversible Zn deposition–dissolution reaction can be kept over 1000 cycles. Such excellent electrochemical performance is due to the stable electrolyte configuration and reinforced interface.

### Deposition Morphology and Chemical Composition Analysis of the Anode Surface

EDS was used to elucidate the distribution of a variety of elements (C, N, O, S) on the Zn surface after cycling (Fig. 4a). The homogeneous distribution of these elements validates that the zinc surface is uniformly armed by the functional groups (–NH_2_, –SO_3_^−^, etc.) of the polymer side chains, which can help guiding the even deposition of zinc ions. XPS analysis was carried out to determine the surface composition of Zn anode which was synergistically functionalized with polyzwitterionic groups and covered by a thin reduction layer after cycles (Figs. 4b and S19). XPS spectra reveal the existence of S_2*p*_ (168.6 eV) and N_1*s*_ (400.15 eV), which can be ascribed to the negatively charged sulfobetaine sulfonate anion (-SO_3_^−^) and the positively charged sulfobetaine ammonium nitrogen (–N^+^(CH_3_)_2_CH_2_). The other peak of element N, at 402.5 eV, may be attributed to the –NH_2_ groups. The C_1*s*_ core level XPS spectra could be fitted into three peaks at 284.8, 286.3 and 288.7 eV, which can be assigned to carbon atoms of saturated hydrocarbons (C–C), carbon atom bonding to oxygen (C–O), sulfur (C–S) or nitrogen (C–N) and carbon atoms in carbonyl groups (C=O), respectively. Interestingly, according to high-resolution XPS spectra of Cl_2*p*_, the Cl_2*p*3/2_ and Cl_2*p*1/2_ peaks at 199.1 and 200.7 eV can be ascribed to Cl^−^, suggesting that the reduction of ClO_4_^−^ formed a thin interlayer on the Zn anode. Integrated with the Zn_2*p*_ characteristic peaks of Zn–O (1019.5 eV), the formation of polyzwitterion-functionalized and Cl^−^ containing layer could synergistically stabilize Zn electrochemistry. Although Cl_2*p*_ peaks also appear in the XPS spectra of anode cycled in zinc perchlorate liquid electrolyte (Fig. S20), the severe zinc dendrites and chaotic morphology formed in the absence of the organic promoting layer greatly shortened the lifetime of liquid electrolyte-based cells (Figs. S11 and 3a–d). Moreover, to investigate the stability of the organic-facilitated layer, the interlayer on the anode surface after cycling was manually peeled off and collected for infrared spectroscopy analysis. In general, the position of the infrared characteristic peak does not change significantly, indicating that the gel electrolyte does not undergo any decomposition or other reactions after electrochemical cycling, which is important for the unceasing manipulation of dendrite-free Zn (Fig. 4c). However, the slight change in peak intensity may be caused by the changed bonding environment under a current field. The broadening of the peak at 3500 cm^−1^ (–OH) and the appearance of inorganic peaks below 500 cm^−1^ (Zn–O) are induced by the water-induced passivation. Meanwhile, the peak near 2019 cm^−1^ which corresponds to the vibration absorption peak of –NH_3_^+^, may be attributed to the protonation process such as water-related reaction in the acidic environment during cycling. Meanwhile, the EIS spectrum of Zn symmetric cell with ADC-gel electrolyte after cycling for 3000 h was recorded. The reduced interfacial impedance demonstrates the optimization of zinc plating/stripping kinetics and the cycling stability of the anode (Fig. S14). It is widely acknowledged that, in aqueous ZMBs, rampant side reactions on the anode are regarded as the dominant factor that leads to cell failure. Therefore, the corrosion resistance of the prepared gel electrolyte was plausibly studied using Tafel tests (Fig. S21). Comparing the corrosion behavior of 2 M Zn(ClO_4_)_2_ electrolyte and gel electrolyte (ADC-gel), it shows that the gel one has higher corrosion voltage (0.051 V) but weaker corrosion current (4.487 μA) than the aqueous one, indicating not only better corrosion resistance but much lower corrosion rate (i.e., the anode in the ADC-gel electrolyte is less likely to be corroded). Chemical reaction-induced corrosion pits exposes more Zn matrix to the unfavorable medium-2 M Zn(ClO_4_)_2_ aqueous electrolyte, thus intensifying the corrosion process. Chronoamperometry was selected to analyze the Zn deposition behavior by measuring the current time dependence for the diffusion-controlled process on the Zn anode. Under a constant potential (200 mV), the big variation in current density vs. time (blue curve in Fig. S22) indicates the unrestrained 2D diffusion and rough deposition procedure for over 300 s, owing to the “tip effect” for charge transfer in liquid electrolyte. For the ADC-gel electrolyte, the initial nucleation and planar diffusion occur within 30 s and are followed by an unchanged curve at a lower density of 13 mA cm^−2^, indicating fewer Zn dendrite forms. Namely, due to the zwitterion structure, the ADC-gel electrolyte can strongly adhere to the surface of Zn and provides a constrained diffusion path for Zn^2+^ ions and then the Zn ions are reduced locally, which finally contributes to the finer nucleation and formation of even deposition and dense layer.

**Fig. 5:**
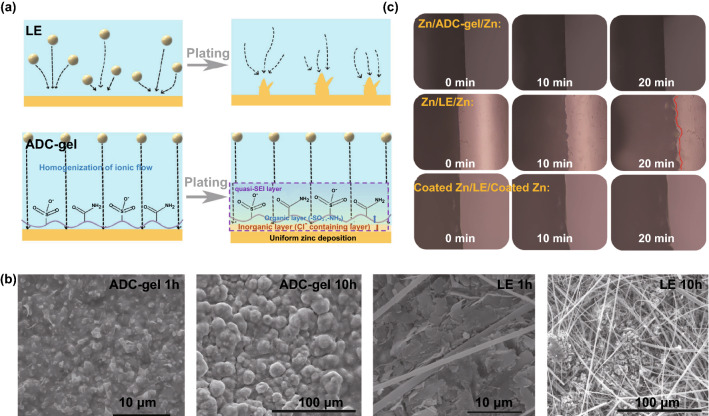
**a** Schematic illustration of the Zn deposition behavior on the anode surface in different electrolytes. **b** SEM images of the deposition of 1 and 10 h Zn on Cu foils in ADC-gel and liquid electrolytes at the current density of 1 mA cm^−2^. **c** In situ optical microscope observing the Zn deposition at a voltage of 4 mV in Zn//Zn symmetric cells to investigate the validity of SEI layer

The interfacial Zn deposition behaviors in different electrolytes were further revealed and are schematically illustrated in Fig. 5a. In the liquid electrolyte, Zn deposition tends to evolve into dendrites which is thermodynamical and kinetical favorable. Conversely, the gel electrolyte with charged and polar functional groups (–SO_3_^−^ and –NH_2_) is expected to guide the migration of Zn ion and facilitates the formation of a dendrite-free surface. The advantageous deposition behavior of the ADC-gel electrolyte could be further investigated by controlling the deposition time in Zn//Cu asymmetrical batteries. The optical images of the disassembled Cu substrate after electrodeposition elucidate a uniform deposition in the gel electrolyte but a preferential and aggregate deposition in the stronger electric field in the liquid electrolyte (Fig. S23). As shown in Fig. 5b, substantial micron-grade Zn sheets with sharp edges are observed on the Cu substrate after 1 hour deposition in the liquid electrolyte. The continuous Zn aggregations evolve to bigger and chaotic clusters, and even larger quantity of pulverized and crushed structure also occurs with the deposition time extending to 10 h. The gel electrolyte, however, on the contrary, exhibits a uniform deposition with close-grained zinc plates, which again verifies the fine nucleation in the gel electrolyte. As the deposition time increases to 10 h, the surface of the Cu substrate represents a hilly like integrated morphology while without any pulverization and clusters, which is mainly attributed to the constrained ion transfer channel of the polyzwitterion structure. In situ optical microscope observation provides intuitive evidence for the validity and the stability of the Zn plating behavior modulated by the quasi-SEI in homemade cells (symmetric Zn//Zn cells with the same width electrode in different electrolytes). As shown in Fig. 5c, the uniform and flat Zn deposition can be clearly observed in the ADC-gel electrolyte-based cell at the current of 4 mA within 20 min. In stark contrast, a visibly uneven Zn deposition morphology with irregular bumps occurred in the liquid electrolyte under the same current density. To further visualize the dynamic stability of the quasi-SEI layer on the Zn anode, the Zn anodes, which had been cycled in the ADC-gel electrolyte, were reassembled into the symmetric cell with liquid electrolyte. Within 20 min, interestingly, flat deposition instead of rough protrusions appeared. This profits from the homogenization ion flux induced by the tightly adhered hydrogel on the anode surface, demonstrating the effectiveness and significance of the surface-modified Zn anodes in liquid electrolytes. Thus, the synergetic quasi-SEI layer can produce several functions: (1) the electrically insulating but ionically conductive inorganic inner layer can not only create a relative water-free microenvironment on the anode surface in inhibiting the successive corrosion, but also construct a fast ion channel with tiny Zn^2+^ transport resistance in lowering overpotential. (2) the zwitterionic-facilitate organic outer layer can guide the homogeneous distribution of Zn^2+^ ion on the interface through the ion confinement effect, resulting in superior electrochemical performance even at high current density. Hence, the composite SEI formed in ADC-gel electrolyte is far more compatible than that formed in the liquid electrolytes, resulting in excellent zinc electrochemistry and ultra-long cycling lifetime of batteries.

### Electrochemical Performance of Zn//MnO_2_ Cells Cycled with ADC-gel Electrolyte

**Fig. 6:**
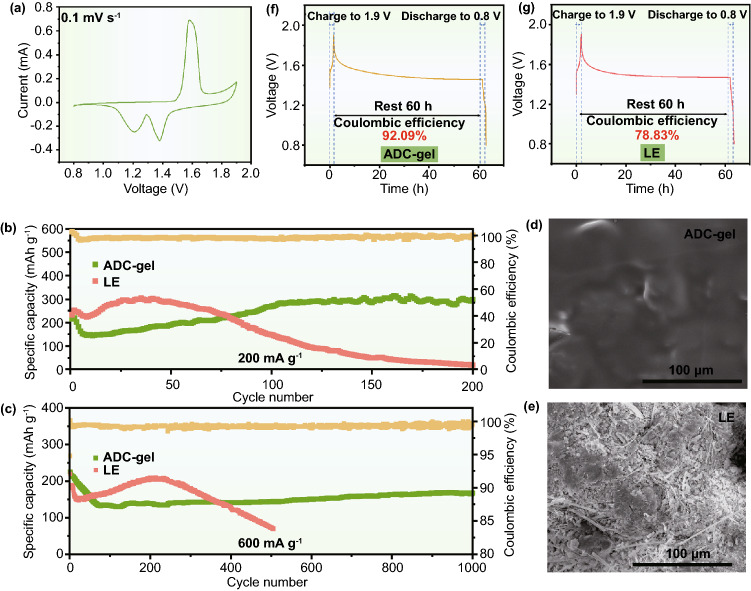
**a** CV curve of Zn//MnO_2_ battery with ADC-gel electrolyte. The cycling performances and the corresponding CE of ADC-gel and liquid electrolytes at a current density of **b** 200 mA g^−1^ and **c** 600 mA g^−1^. SEM images of anode after cycles in **d** Zn/ADC-gel/MnO_2_ and **e** Zn/LE/MnO_2_ batteries under the current density of 200 mA g^−1^. The self-discharge tests performed on **f** ADC-gel electrolyte and **g** liquid electrolyte by fully charged after 10 cycles at 200 mA g^−1^ and then shelving for 60 h

The quasi-solid-state Zn//MnO_2_ batteries were assembled by replacing the conventional membrane with the prepared ADC-gel electrolyte to further evaluate the electrochemical performance. The MnO_2_ cathode materials synthesized by a simple hydrothermal method could be well indexed to MnO_2_ (JCPDS No. 72–1982), as shown in Fig. S24. CV was employed to manifest the battery behaviors. As shown in Figs. 6a and S25, the curves reveal similar typical redox reactions at the scan rate of 0.1 mV s^−1^ in ADC-gel and liquid electrolytes. Two reduction peaks at 1.2 and 1.38 V are ascribed to the formation of MnOOH (1.38 V) and the transformation from Zn to Zn^2+^ (1.2 V), respectively. The overlapped oxidization peak at 1.58/1.62 V corresponds to the regenerating of MnO_2_ and the zinc plating. At a small current density of 200 mA g^−1^ (Fig. 6b), although the curve of the gel electrolyte-based cell (in green) shows a capacity rise in the first 100 cycles due to the wetting process of cathode materials [66], it can keep stable more than 100 cycles with a high capacity of 300 mAh g^−1^. In contrast, the capacity of liquid electrolyte decays immediately after a shorter capacity climbing. Both batteries show a decrease in capacity in the first few cycles, which may be caused by the instability of the interface [67]. As shown in Fig. 6d–e, the corresponding morphology of the Zn anode harvested from the Zn//MnO_2_ cell with gel electrolyte after cycling at the current density of 200 mA g^−1^ is still very flat and dense. Conversely, the SEM image of the anode surface cycled in the liquid electrolyte is overgrown with rampant dendrite and by-products. The battery using ADC-gel electrolyte can also undergo a higher current density (600 mA g^−1^) and deliver a relatively high capacity of 150 mAh g^−1^ with more than 1000 stable cycles (Fig. 6c). Meanwhile, the high CE (nearly 100%) of full cells with ADC-gel electrolytes further demonstrate the superiority of the gel electrolyte. Due to the existence of inevitable self-discharge and self-corrosion phenomena of the aqueous ZIBs, the shelving and restoring mode reported before [68] was introduced to further verify the superiority of the ADC-gel electrolyte. The batteries were assembled and fully charged to 1.9 V after 10 cycles, then shelved for 60 h and end up with the full discharging to 0.8 V. It can be seen that after being left for 60 h, the CE of the ADC-gel electrolyte can reach 92.09% (Fig. 6f), while the liquid electrolyte only maintains 78.83% (Fig. 6g). Thus, the evidence discussed above confirms the ADC-gel is an important driving factor for a long shelf-life battery. As we have known, the interface side reactions on the metal anode and the dissolution of the cathode materials are the main limitations for long shelf life and favorable restoration capacity. Although the in situ reduction layer on the surface of Zn metal can hinder the unwanted reactions with active water to a certain degree in the liquid electrolyte, the layer is, however, not as compatible as the one generated in the gel electrolyte, which means rampant parasitic reactions still exist. Meanwhile, untrammeled ion and electron movement in the liquid electrolyte together with the by-products induced by the side reactions can enlarge the polarization voltage, deteriorate self-discharge and cause the battery failure after only a few cycles. In contrast, the synergy of the inorganic inner layer and the organic outer layer of the ADC-gel electrolyte can greatly suppress the pernicious self-discharge on the interface. In other words, the uniform and dense insulating layer dynamically contacted with the zinc metal can inhibit the self-corrosion of the Zn anode. The charged groups (–SO_3_^−^ and N^+^) of the polyzwitterionic hydrogel can tether the ions and restrain their self-diffusion, thereby slowing and reducing side reactions. The continuous and detrimental exhaustion of the active zinc metal can be alleviated remarkably in the gel electrolyte during shelving. In addition to MnO_2_, vanadium pentoxide is another promising cathode material for aqueous ZMBs. Cathode materials were prepared using commercialized V_2_O_5_ as raw material. It is impressive that the long cycle performance and CE of the V-based full cell with ADC-gel electrolyte are prominent. As shown in Fig. S26**,** at the current density of 1 A g^−1^, the ADC-gel-based full cell keeps stable cycling with a high specific capacity of 400 mAh g^−1^ and the CE of 99.8%. Conversely, the specific capacity of LE-based full cell decays immediately and the CE fluctuates apparently after more than 100 cycles. Accordingly, ADC-gel electrolyte can also effectively inhibit the dissolution of vanadium-based active materials and promote their stable cycling, demonstrating its wide applicability on cathode materials.

**Fig. 7:**
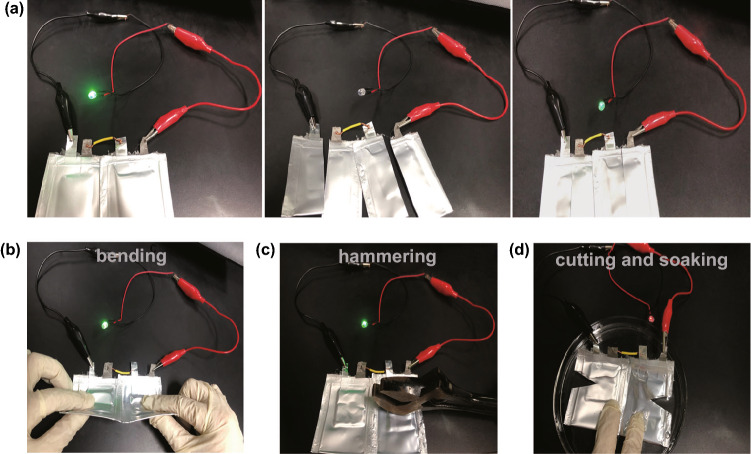
Optical images of destructive tests of the two pouch cells in series including **a** cutting and reconnecting test, **b** bending test, **c** hammering test and **d** cutting and soaking test

The excellent flexibility and mechanical properties enable the ADC-gel electrolyte a promising future for the application of wearable devices. The flexible MnO_2_/ADC-gel/Zn batteries were assembled to power a light-emitting diode (LED) (Fig. 7). The destructive test by cutting and reconnecting batteries is shown in Fig. 7a. When the flexible batteries were completely cut into halves, the LED turned off immediately, but it lit up immediately after reconnecting the severed batteries, showing the impressive anti-vandalism and liquid leakage prevention feature of the ADC-gel electrolyte. Notably, the flexible quasi-solid-state battery can still work even when subjected to harsh external forces such as bending and hammering (Fig. 7b–c). The electronic device can be driven normally even obvious creases and pits appear after multiple bending and hammering. The impressive tailoring ability can be demonstrated by the cutting test. As shown in Fig. 7d, after cutting pouch cells many a time, LED can still be turned up by the ZMBs even when exposed to a dish of water. Such behavior of the ZMB makes it easy to customize into various shapes and patterns without a complicated preparation process and possess a certain degree of water resistance. The flexible ZMBs can withstand extreme conditions such as bending, hammering, cutting and soaking as well. The superior properties discussed above demonstrate the battery with ADC-gel electrolyte may be a promising candidate for advanced wearable energy storage devices.

## Conclusions

In summary, the ADC-gel electrolyte synthesized by free radical polymerization exhibits remarkable compatibility toward both the Zn anode and the cathodes (MnO_2_ and V_2_O_5_). The integration of zwitterion-facilitated gel matrix and Zn(ClO_4_)_2_ salt establishes the fast Zn ion transportation channel and ultra-stable Zn electrochemistry. The Zn/ADC-gel/Zn symmetrical cells deliver ultra-long cycling life of 3000 h with relatively low polarization potential (~ 0.03 V) at the current density of 0.5 mA cm^−2^ even after shelving for 120 h. The tolerance of the gel electrolyte with a high current density of 5 mA cm^−2^ is also impressive. Such brilliant performance is attributed to the construction of a robust composite quasi-SEI layer composed of Cl^−^ containing inorganic layer and organic layer with ion confinement effect. Under the synergistic effect of the selected gel and salt, the Zn/ADC-gel/Cu cells could cycle for more than 1000 cycles with a laudable average CE of 99.18%. For full cells matched with MnO_2_, the exceptional capacity retention and shelving ability in ADC-gel electrolyte shed light on the remarkable electrochemical performance of this aqueous ZMB. Furthermore, the assembled pouch cells demonstrates impressive waterproof property and excellent resistance toward external forces. The quasi-solid-state ZMBs that integrate excellent mechanical–environment durability and outstanding electrochemical performance can be served as safe, scalable and promising flexible electronics.

## Supplementary Information

Below is the link to the electronic supplementary material.

Supplementary file1 (PDF 1449 KB)
