# Supplementary material for: Building Ultra-Stable and Low-Polarization Composite Zn Anode Interface via Hydrated Polyzwitterionic Electrolyte Construction
Source: Nanomicro Lett. 2022 Apr 6;14:93. doi: 10.1007/s40820-022-00835-3 (PMC8986915; doi:10.1007/s40820-022-00835-3)
Supplement: Supplementary file 1 — Supplementary file1 (PDF 1449 KB) [file 40820_2022_835_MOESM1_ESM.pdf]

Supporting Information for

Building Ultra-Stable and Low-Polarization Composite Zn Anode Interface via Hydrated Polyzwitterionic Electrolyte Construction

Qiong He[1], Guozhao Fang[1], Zhi Chang[1], Yifang Zhang[2], Shuang Zhou[1], Miao Zhou[1], Simin Chai[1], Yue Zhong[1], Guozhong Cao[3], Shuquan Liang[1], and Anqiang Pan[1,] *

[1]School of Materials Science and Engineering, Key Laboratory of Electronic Packaging and Advanced Functional Materials of Hunan Province, Central South University, Changsha 410083, P. R. China

[2]Joint School of National University of Singapore and Tianjin University International Campus of Tianjin University Binhai New City, Fuzhou 350207, P. R. China

[3]Department of Materials Science and Engineering, University of Washington, Seattle, WA, 98195, USA

*Corresponding author. E-mail: pananqiang@csu.edu.cn (Anqiang Pan)

Supplementary Figures

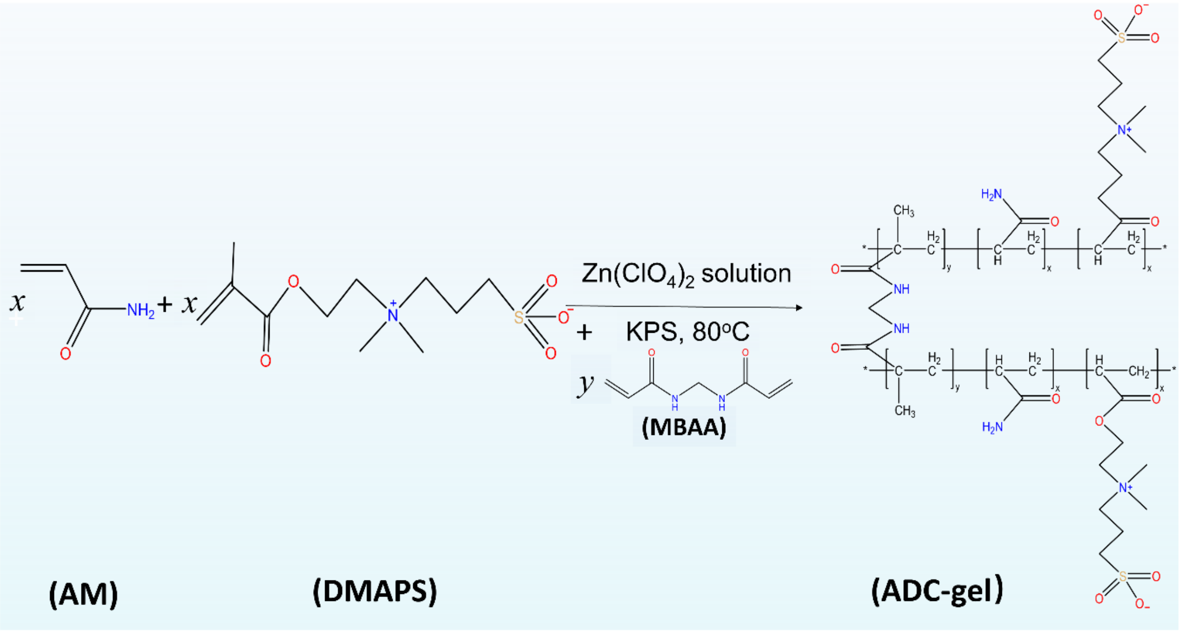

Fig. S1 The mechanism of the radical reaction between AM and DMAPS monomers

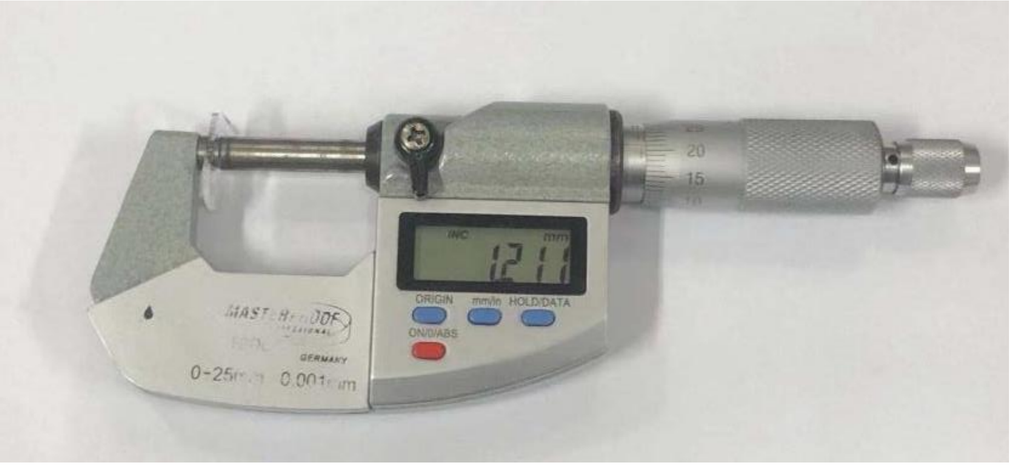

Fig. S2 The thickness optical image of the ADC-gel electrolyte

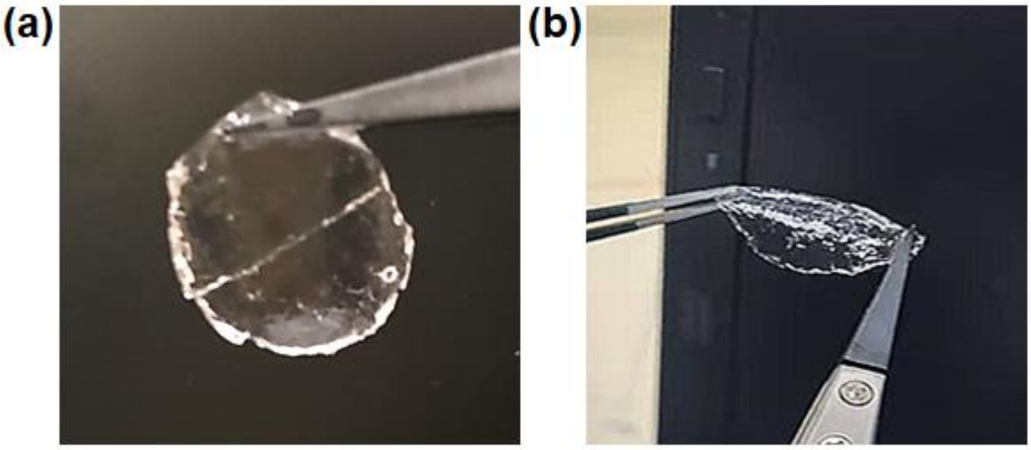

Fig. S3 Optical images of the hydrogel **a** self-healing after a few minutes after two pieces were cut and **b** the stretchability of the two reconnected pieces

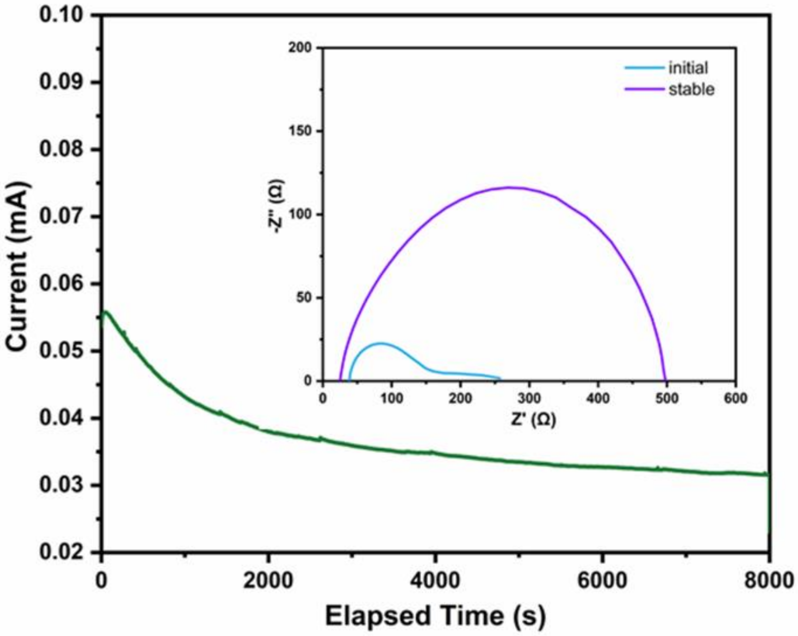

Fig. S4 EIS spectra and the current variation with the polarization of Zn/LE/Zn symmetric cell

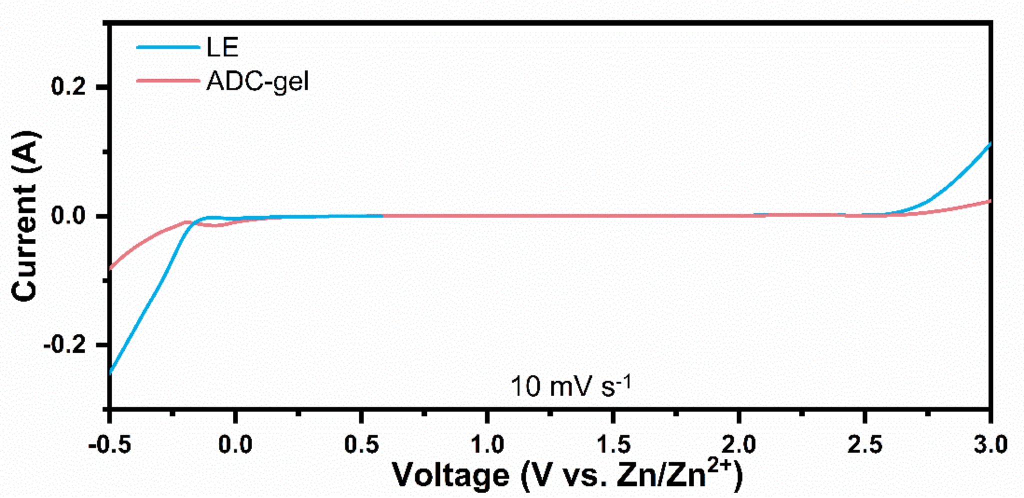

Fig. S5 LSV curves of Zn//stain steel cells at the scan rate of 10 mV s^{-1}

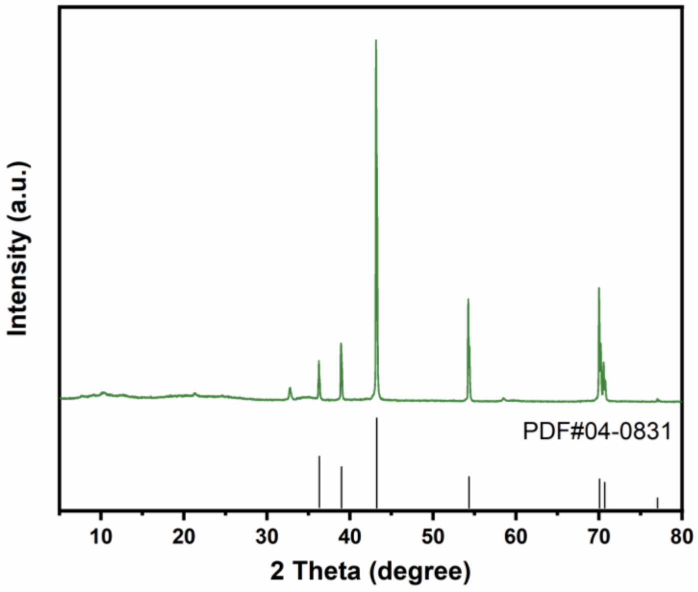

Fig. S6 XRD patterns of the Zn anode cycled in the ADC-gel electrolyte at 0.5 mA cm^{-2} and 0.5 mAh cm^{-2}

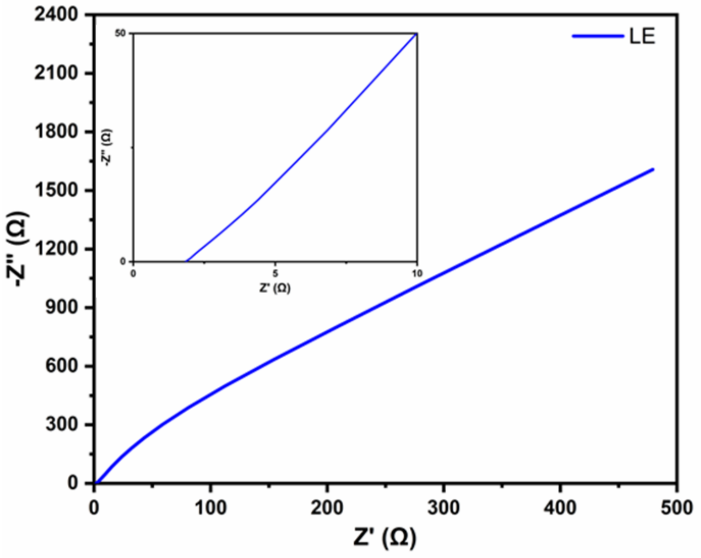

Fig. S7 EIS spectra of the LE with two blocked electrodes (stain steels)

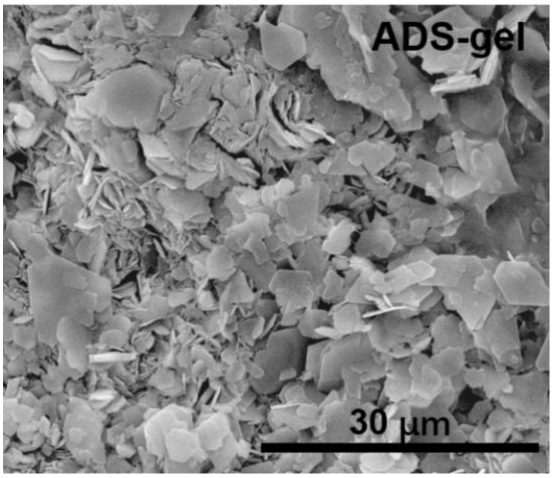

Fig. S8 SEM image of the ADS-gel electrolyte cycled in Zn//Zn symmetrical cell after 50 cycles at the current density of 0.5 mA cm^{-2}

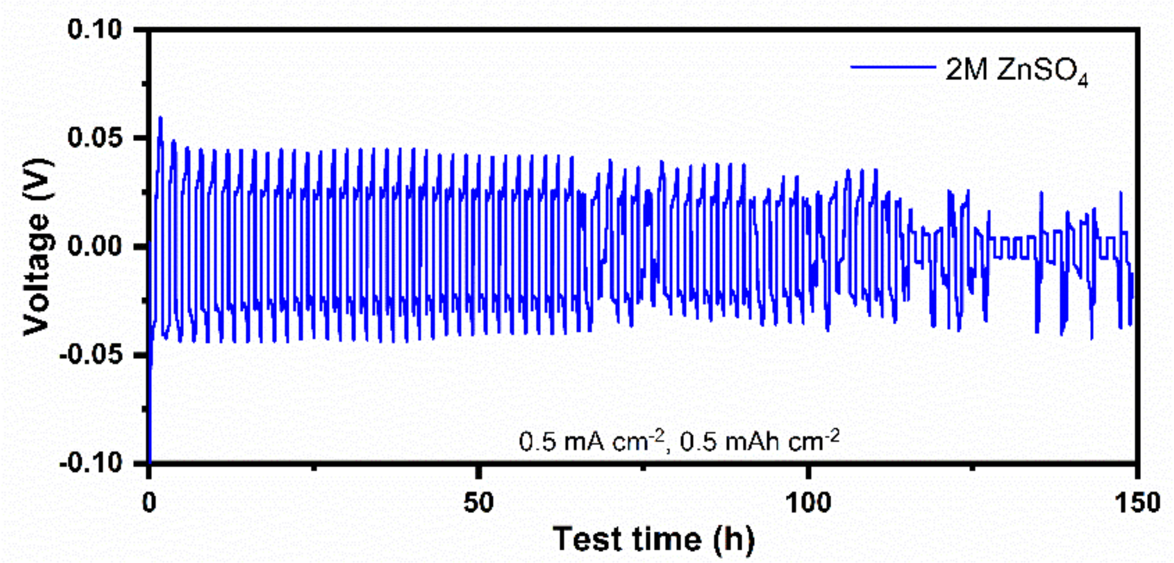

Fig. S9 Cyclic stability of symmetrical cells assembled with 2M $ZnSO_4$ liquid electrolyte under current densities and capacities of 0.5 mA cm^{-2}, 0.5 mAh cm^{-2}

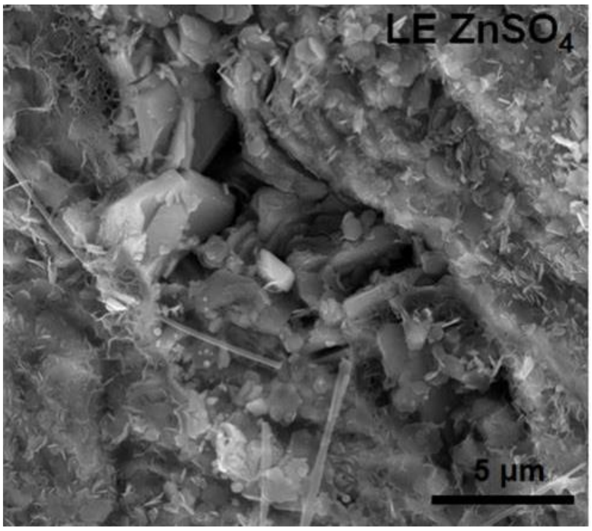

Fig. S10 SEM image of the 2M $ZnSO_4$ aqueous electrolyte cycled in Zn//Zn symmetrical cell at the current density of 0.5 mA cm^{-2}

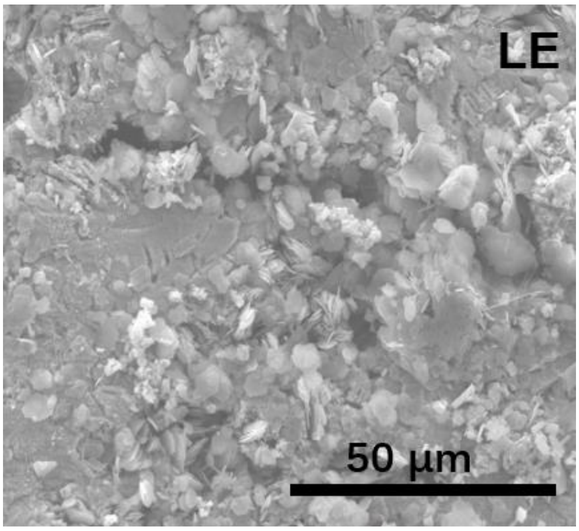

Fig. S11 SEM image of the liquid electrolyte cycled in Zn//Zn symmetrical cell after 60 cycles at the current density of 0.5 mA cm^{-2}

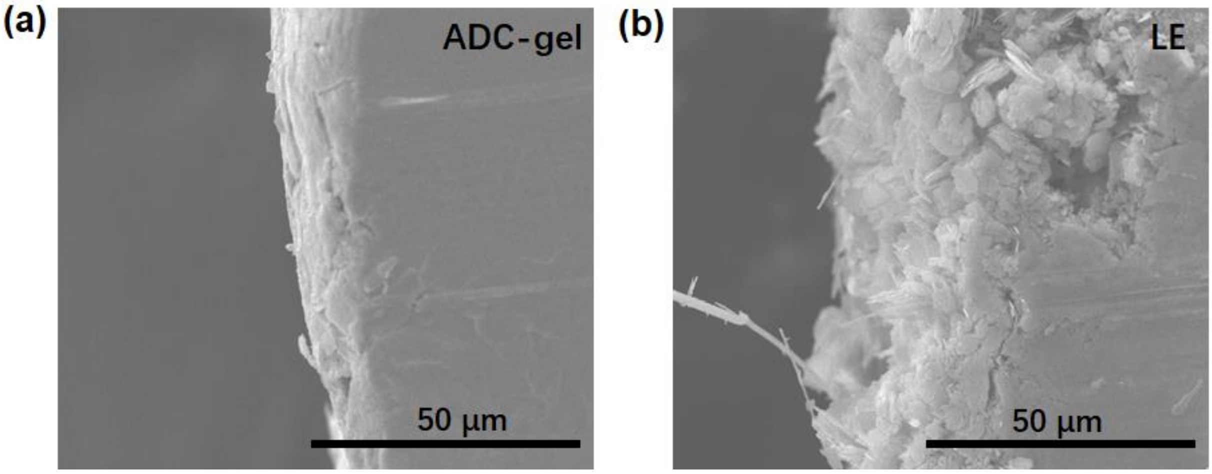

Fig. S12 Cross-section SEM images of Zn anodes in Zn//Zn symmetrical cells after 60 cycles at 0.5 mA cm^{-2} and 0.5 mAh cm^{-2}

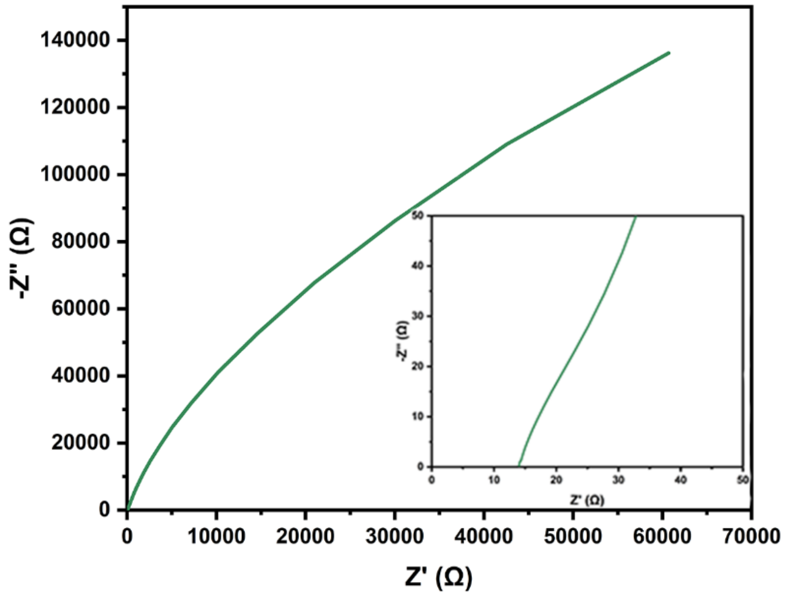

Fig. S13 EIS spectra of the ADS-gel with two blocked electrodes (stain steels)

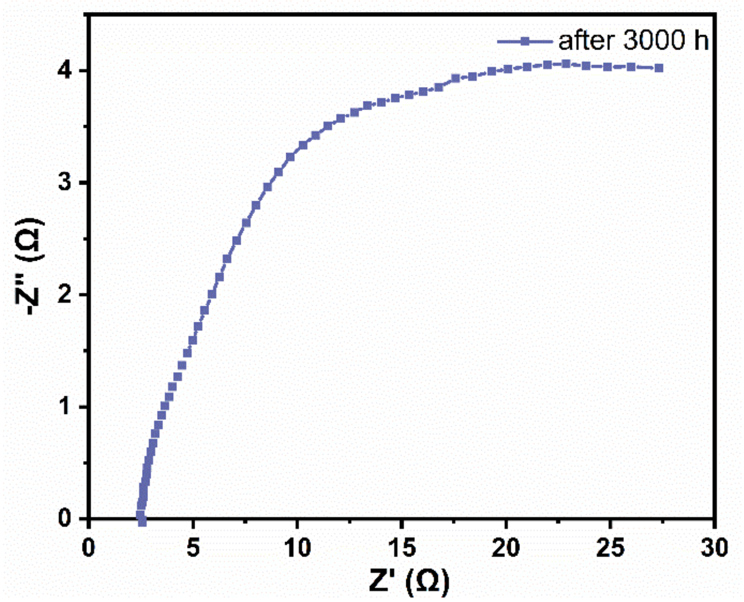

Fig. S14 EIS spectra of Zn/ADC-gel/Zn symmetric cell after cycling 3000 h

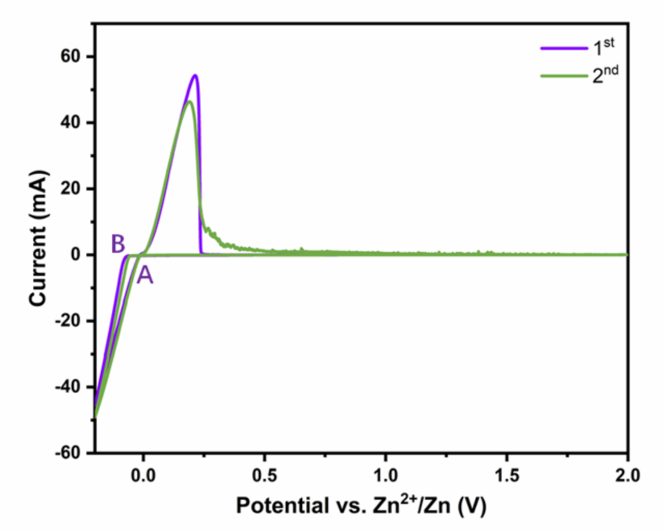

Fig. S15 CV curves of Zn anode dissolution and deposition in Zn/LE/stain steel cell at the scan rate of 0.5 mV s^{-1}

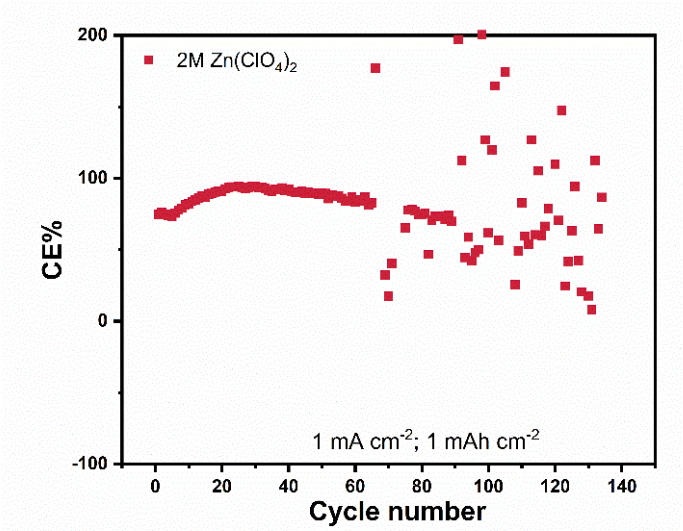

Fig. S16 CE curve of liquid electrolyte in Zn//Cu asymmetric cell

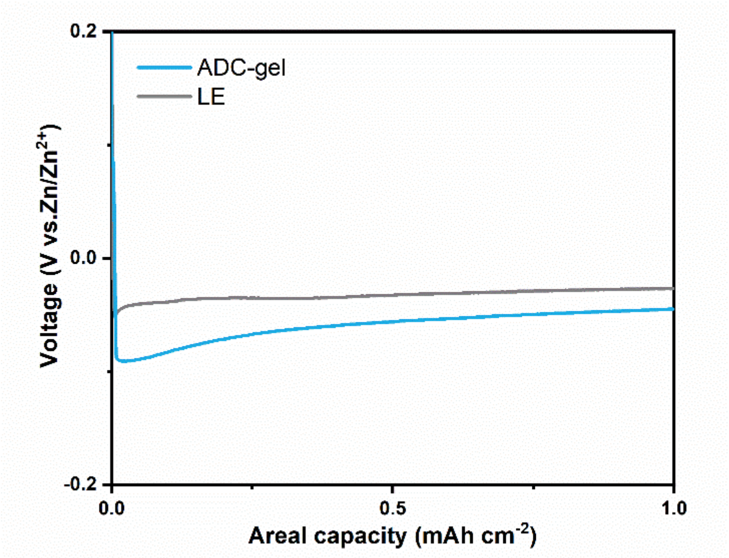

Fig. S17 Nucleation overpotentials of ADC-gel electrolyte and liquid electrolyte in Zn//Cu asymmetrical batteries

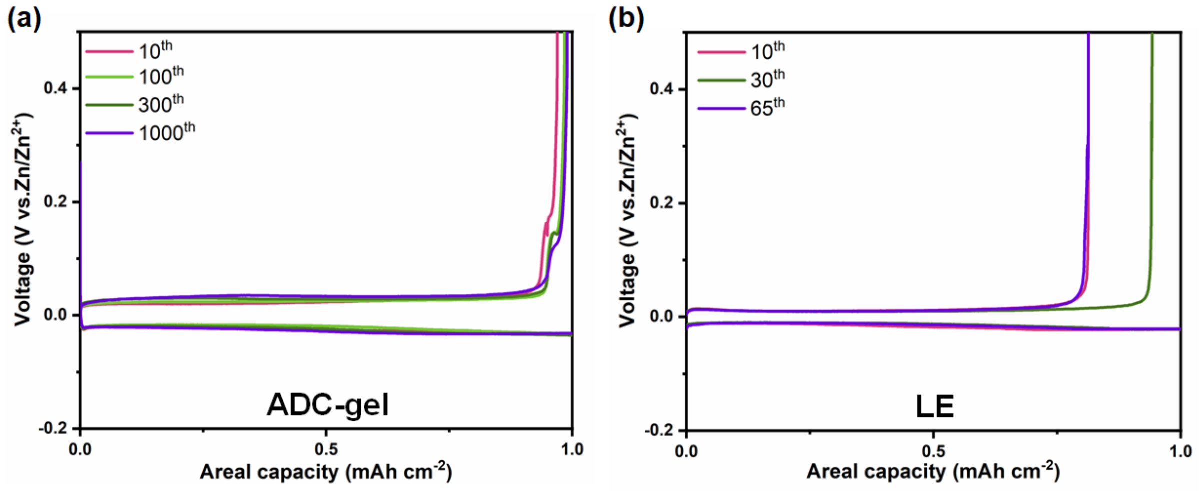

Fig. S18 Voltage profiles of the asymmetrical batteries in ADC-gel and liquid electrolytes

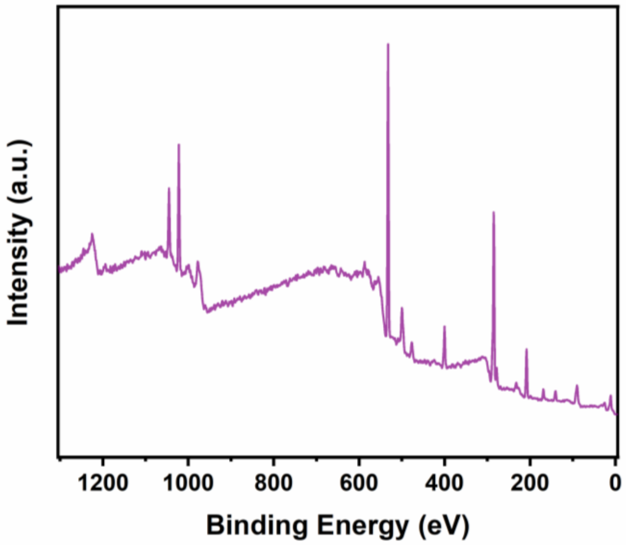

Fig. S19 XPS spectra of the Zn anode cycled in the ADC-gel electrolyte

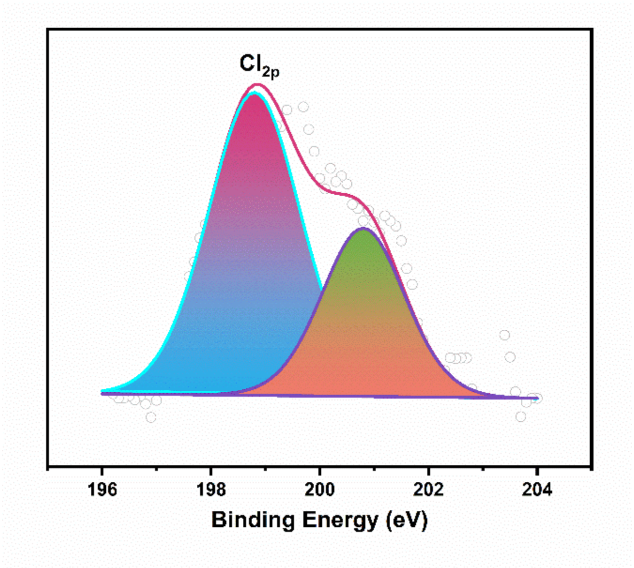

Fig. S20 Cl_{2p} XPS spectra of the surface of the Zn anode cycled in the zinc perchlorate liquid electrolyte

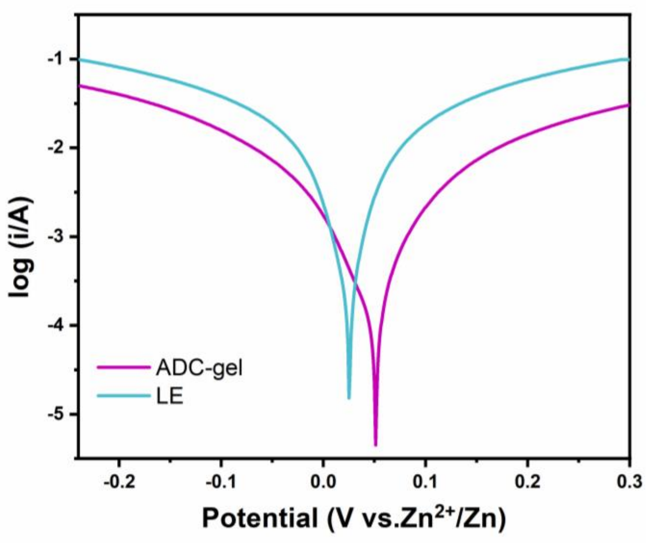

Fig. S21 Tafel curves of the Zn//Zn cells with the ADC-gel electrolyte and the liquid electrolyte

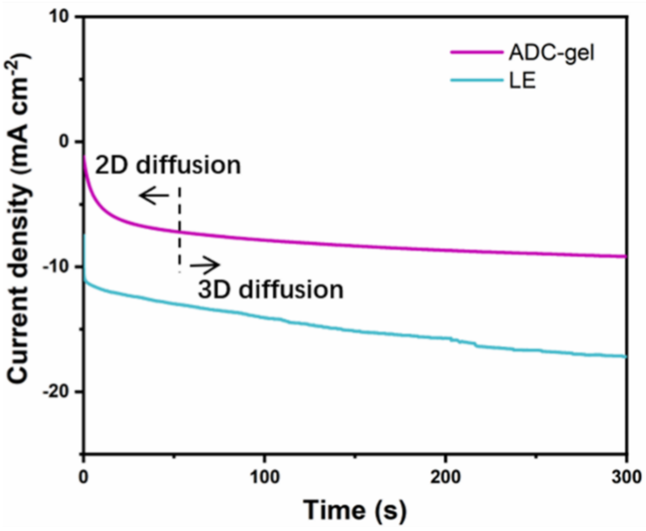

Fig. S22 Chronoamperometry curves of the Zn//Zn cells with the ADC-gel electrolyte and the liquid electrolyte

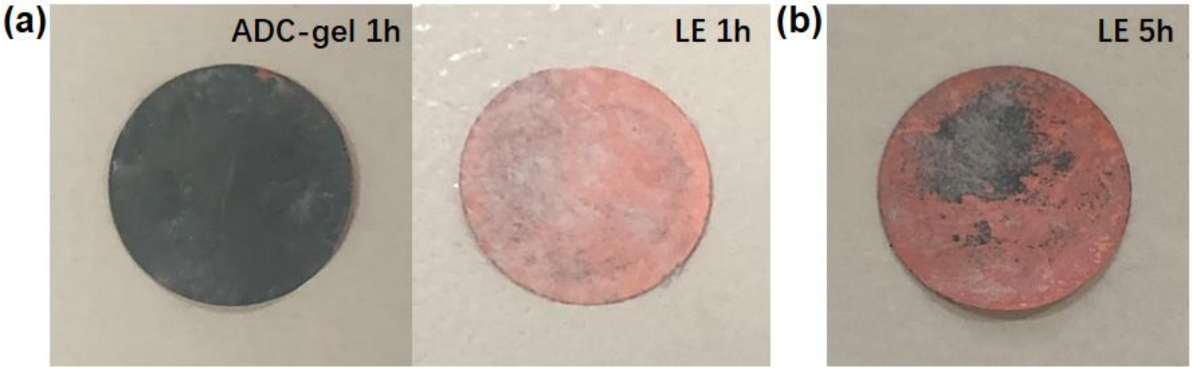

Fig. S23 Optical images of the Zn deposition on Cu foils **a** in ADC-gel and liquid electrolyte for 1 hour and **b** in liquid electrolyte for 5 hours

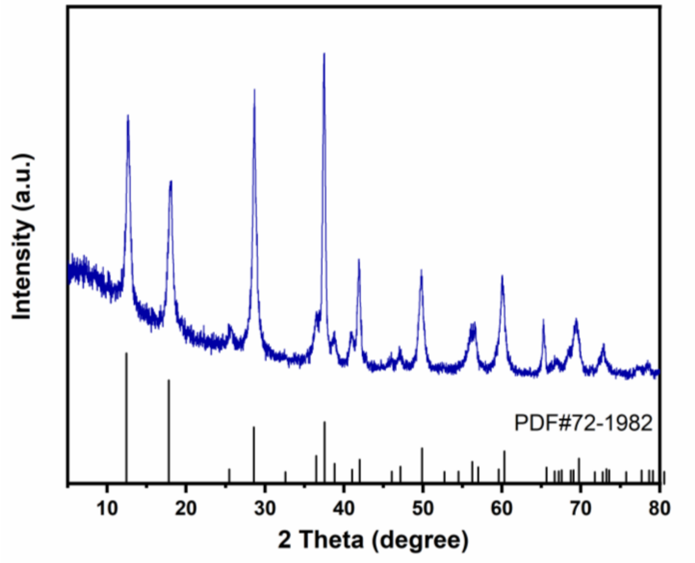

Fig. S24 XRD pattern of the prepared MnO_2 cathode by traditional hydrothermal method

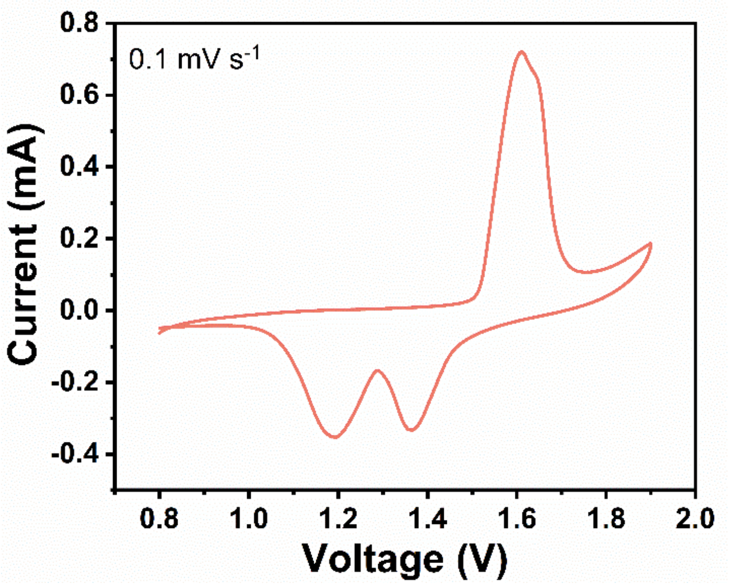

Fig. S25 CV curve of Zn//MnO_2 battery with the liquid electrolyte

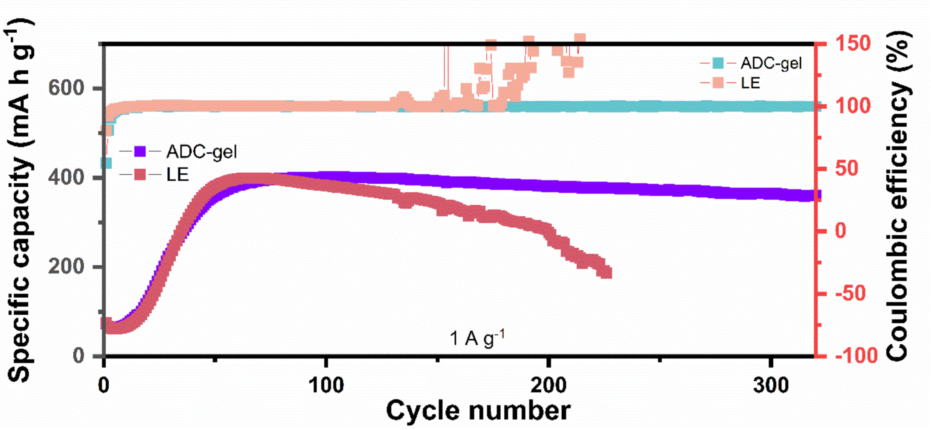

Fig. S26 The cycling performances and the corresponding CE of Zn//V_2O_5 full cells with different electrolytes at a current density of 1 A g^{-1}

Table S1 Comparison of polarization voltage and cycle life of hydrogels

hydrogels	Current density (mA cm^{-2})	Polarization voltage (mV)	Cycle life (h)
PAM (3M $Zn(CF_3SO_3)_2$) [S1]	0.2	50	500
ZSC-gel [S2]	0.5	85	200
Alg-Zn [S3]	1.77	100	270
HGE2M [S4]	0.5	85	1000
GG/SA/EG [S5]	0.2	40	200
CT3G30 [S6]	2	65	800
ADC-gel (this work)	0.5/1/5	30/38/50	3000/1700/650

Supplementary References

[S1] W. Deng, Z. Zhou, Y. Li, M. Zhang, X. Yuan et al., High-capacity layered magnesium vanadate with concentrated gel electrolyte toward high-performance and wide-temperature zinc-ion battery. ACS Nano **14**(11), 15776–15785 (2020). https://doi.org/10.1021/acsnano.0c06834

[S2] F. Mo, Z. Chen, G. Liang, D. Wang, Y. Zhao et al., Zwitterionic sulfobetaine hydrogel electrolyte building separated positive/negative ion migration channels for aqueous Zn-MnO_2 batteries with superior rate capabilities. Adv. Energy Mater. **10**(16), 2000035 (2020). https://doi.org/10.1002/aenm.202000035

[S3] Y. Tang, C. Liu, H. Zhu, X. Xie, J. Gao et al., Ion-confinement effect enabled by gel electrolyte for highly reversible dendrite-free zinc metal anode. Energy Storage Mater. **27**, 109–116 (2020). https://doi.org/10.1016/j.ensm.2020.01.023

[S4] X. Lin, G. Zhou, J. Liu, M. Robson, J. Yu et al., Bifunctional hydrated gel electrolyte for long-cycling Zn-ion battery with NASICON-type cathode. Adv. Funct. Mater. **31**(42), 2105717 (2021). https://doi.org/10.1002/adfm.202105717

[S5] J. Wang, Y. Huang, B. Liu, Z. Li, J. Zhang et al., Flexible and anti-freezing zinc-ion batteries using a guar-gum/sodium-alginate/ethylene-glycol hydrogel electrolyte. Energy Storage Mater. **41**, 599–605 (2021). https://doi.org/10.1016/j.ensm.2021.06.034

[S6] M. Chen, J. Chen, W. Zhou, X. Han, Y. Yao et al., Realizing an all-round hydrogel electrolyte toward environmentally adaptive dendrite-free aqueous Zn-MnO_2 batteries. Adv. Mater. **33**(9), 2007559 (2021). https://doi.org/10.1002/adma.202007559
